# A Prolapsed Cecoureterocele in an Adult Treated Conservatively: Highly Rare, but Existent

**DOI:** 10.1155/2016/5049072

**Published:** 2016-09-14

**Authors:** Löcherbach Florian, Preusser Stefan, Meier Mark

**Affiliations:** Department of Urology, GZO Hospital Wetzikon, Spitalstrasse 66, 8620 Wetzikon, Switzerland

## Abstract

Ectopic ureteroceles are one of the most common human urinary tract anomalies. They tend to be recognized and treated in early childhood, especially when they have prolapsed. In most cases surgical therapy is inevitable. In this case report, however, we present the unusual case of a 26-year-old woman suffering from a prolapsed cecoureterocele without any known history of an ectopic ureterocele so far. She was successfully treated without the need for a surgical procedure.

## 1. Introduction

With an incidence of 1/5000 to 1/12000 per birth, ureteroceles belong to the most common urinary tract anomalies in humans [1, 2]. Still, they are considered to be rather rare and tend to be diagnosed by pediatricians and less often by urologists who primarily treat adults. Furthermore, according to literature, only 5% of all ureteroceles are likely to prolapse and if they do, they normally do it in early childhood. There are a just a fistful of case reports to be found in literature so far, which describe similar cases to our case at hand, where a cecoureterocele was first diagnosed in a grown-up patient. As an example there is the case of a 59-year-old woman from Turkey published by Simsir et al. [3] or another case of a 24-year-old Mexican woman published by Villagómez-Camargo et al. [4]. In both cases, however, the patient had to undergo surgery in the end, which makes the difference to the case we present hereby.

## 2. Case Presentation

**Figure 1:**
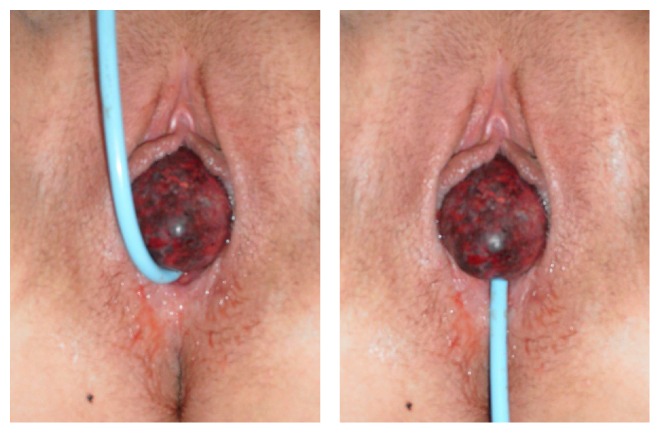
Initial clinical findings, after a transurethral catheter was placed. As will be seen in both pictures, we initially found a prolapsing vaginal mass, whose origin was not to be clearly identified without further examinations.

A 26-year-old woman admitted herself for urological consultation to our hospital. She described a recent history of gross haematuria as well as having recognized a prolapsing vaginal mass. After our first examination we presumed the vaginal mass to be a thrombosed urethral caruncle. The initial sonographic evaluation of both kidneys revealed no pelvic dilatation. While the first try of carefully repositioning our clinical findings (Figure 1) was unsuccessful, we decided to admit the patient to the operating theatre in order to do further examinations and explorations under anesthesia. If the presumed diagnosis of a thrombosed urethral caruncle was correct, deroofing the caruncle would have been possible in the same instance.

**Figure 2:**
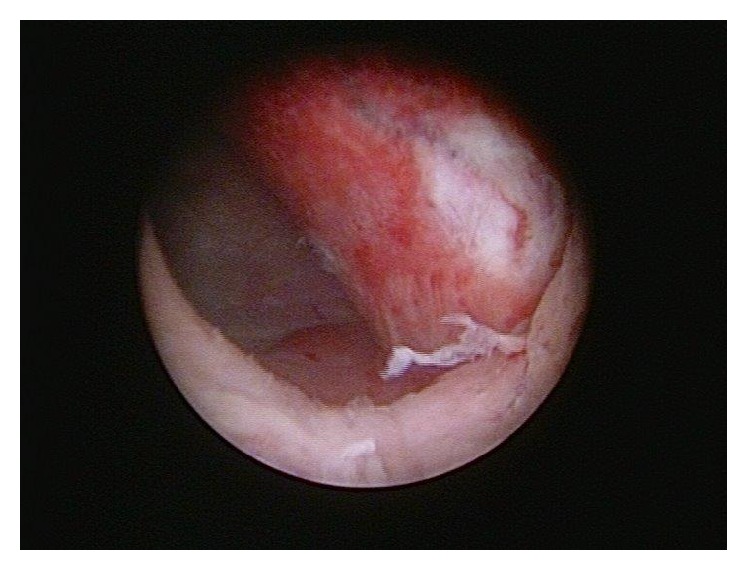
Intraoperative findings during cystoscopy. On the left patient's side the repositioned ureterocele is to be seen. The ureteral orifice could not be identified.

During the examination in general anesthesia we found that some clear fluid was secreted from the circular prolapsed vaginal mass when putting pressure on it. Upon further pressure the mass retreated itself spontaneously into the outer urethral meatus. As will be seen in Figure 2, in the following cystoscopy we found a big ureterocele on the left patient's side, matching the formerly prolapsed mass. The left ureteral ostium was not to be identified.

The right-sided ureteral ostium showed some small protrusion as well, presumably coinciding with another small ureterocele on this side. The further cystoscopy was largely inconspicuous so that we decided to end the procedure due to the now resolved acute situation which, nonetheless, required further investigation.

**Figure 3:**
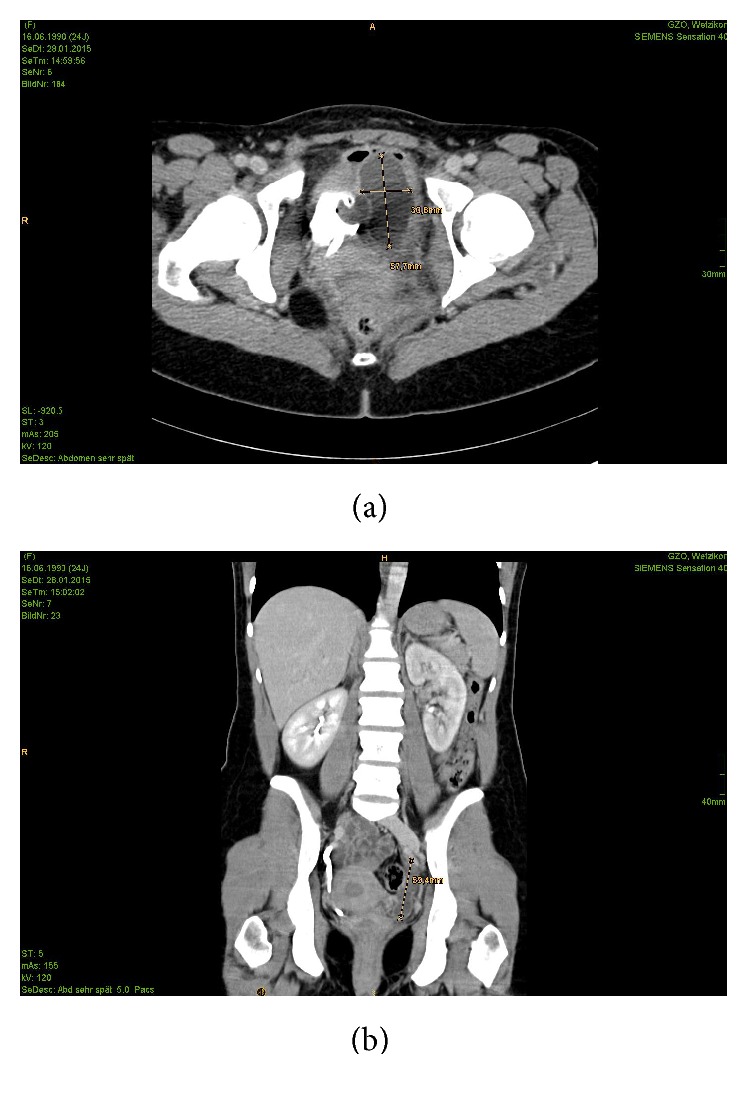
Transversal (a) and frontal (b) section of the patient's CT scan. In (a), the bladder, filled with contrast and a transurethral catheter being in place, can be found shifted to the right patient's side. The ureterocele right next to the bladder measures about 3 × 6 cm. (b) shows the dilated left ureter and that there was no incidence for a duplex renal system.

In order to confirm our intraoperatively newly obtained diagnosis of a prolapsed ureterocele, we did a CT scan of the patient's abdomen the day after the surgical intervention. In the CT scan (Figure 3) the presumed huge ureterocele on the left patient's side was indeed confirmed, accompanied by consecutively dilated left ureter and renal pelvis. There was no incidence for a duplex pelvic system. The right ureter was found to be normal with an inconspicuous ureteral ostium into the bladder.

While being clinically asymptomatic and with the renal retention parameters being always within normal range, the patient was then discharged from hospital and followed up one month later. In the follow-up examination there was no sonographic incidence for a dilated renal pelvis on both sides and in cystoscopy the left-sided ureterocele seemed to have regressed in size. Even the ureteral ostium was now easily identified.

**Figure 4:**
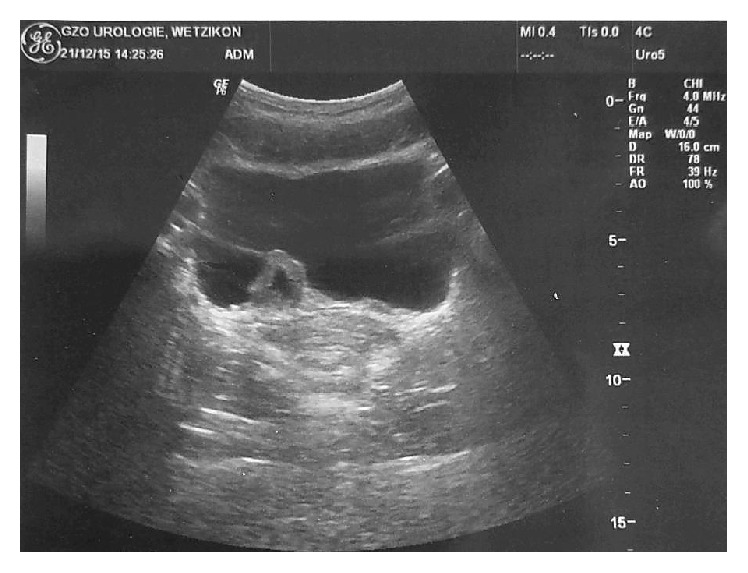
Transversal section of the sonographic control at the 12-month follow-up. The left-sided ureterocele can be seen side-inverted on the picture's right side. Compared to the initial CT scan (Figure 3), there is a significant regression of the ureterocele in size.

**Figure 5:**
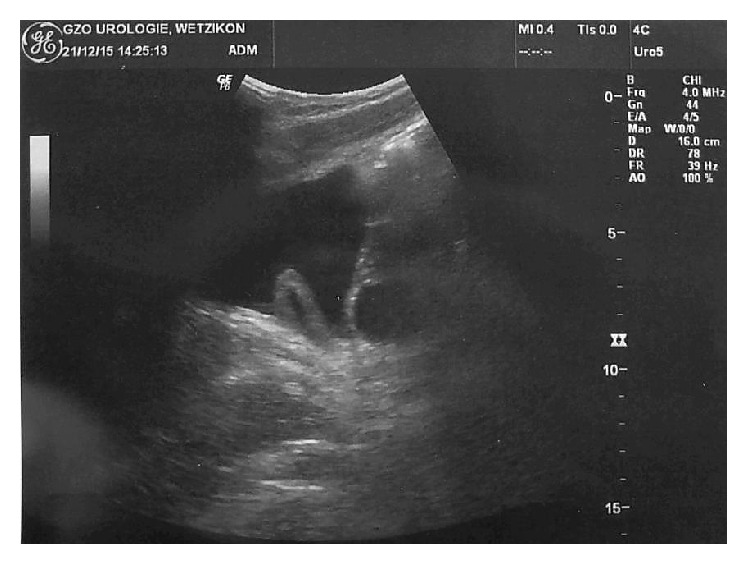
Sagittal section of the sonographic control at the 12-month follow-up. The ureterocele has significantly downsized compared to the initial CT scan (Figure 3).

We established two further follow-up appointments 5 and 12 months after the initial consultation. In both cases the patient was found to be absolutely asymptomatic. No further episodes of gross haematuria or urinary tract infections were reported. Sonography always showed normal conditions concerning the renal drainage and sonography as well as cystoscopy during the 5- and 12-month follow-ups revealed a further regression of the ureterocele in size. The sonographic findings of the 12-month follow-up are to be seen in Figures 4 and 5.

## 3. Discussion

Since a prolapsed cecoureterocele, especially in adults, is a highly rare condition, it is rather difficult to give an evidence-based recommendation upon its treatment. The primary goal however should always be the prevention of complications such as loss of renal function, recurrent urinary tract infections, and urinary incontinence [5]. Due to the anatomically complex situations which have to be faced, when treating a patient suffering from a ureterocele, gaining diagnostic clearance of these circumstances should always be the first step in approaching the patient's treatment. In order to achieve this clearance, radiologic tools such as sonography, CT/MRI-scans, or retrograde ureteropyelography should be chosen for each patient on an individual basis.

In cases of blindly ending ureters, hypoplastic renal segments, which are drained by the ectopic ureter, or infrasphincteric ureteral orifices, a surgical approach such as heminephrectomy and ureterectomy is largely considered to be the first-line therapy [3, 5]. Another approach for less complicated ureteroceles without a duplex pelvic system or hypoplastic renal segments is the endoscopic deroofing and DJ-stenting of the ureterocele [6].

Still our patient was asymptomatic in all follow-up examinations so far, without having ever undergone any surgical intervention except the repositioning of the ureterocele in anesthesia. We therefore suggest that cases of cecoureterocele in adults, which have been asymptomatic in their lives so far, should, after ruling out any complicating circumstances, be treated conservatively in the first place. Close-meshed follow-ups have to be established nonetheless, in order to be able to surgically interfere as soon as there are any complications under the conservative treatment. This strategy is also proposed by the only retrospective study about the topic, which has been published so far [7]. Even when it comes to infants and children, the paradigm of primary reconstruction of the urinary tract in patients suffering from ureterocele has lately started to shift. Less aggressive surgical approaches like endoscopic puncture of the ureterocele or even nonoperative treatment seem to achieve similar functional results [8]. Still, the decision upon the specific treatment of each patient, especially when treating children, should be made considering the individual diagnostic findings at hand.
